# Commissioning of a PTW 34070 large‐area plane‐parallel ionization chamber for small field megavoltage photon dosimetry

**DOI:** 10.1002/acm2.12185

**Published:** 2017-10-04

**Authors:** Tom Kupfer, Joerg Lehmann, Duncan J. Butler, Ganesan Ramanathan, Tracy E. Bailey, Rick D. Franich

**Affiliations:** ^1^ School of Science RMIT University Melbourne Vic. Australia; ^2^ Radiation Oncology Centre Austin Health Heidelberg Vic. Australia; ^3^ School of Science RMIT University Melbourne Vic. Australia; ^4^ Faculty of Science The University of Sydney Sydney NSW Australia; ^5^ Department of Radiation Oncology Calvary Mater Newcastle Waratah NSW Australia; ^6^ Australian Radiation Protection and Nuclear Safety Agency Yallambie Vic. Australia; ^7^ School of Science RMIT University Melbourne Vic. Australia

**Keywords:** dose‐area product, large‐area chamber, linear accelerators, quality assurance, small field dosimetry

## Abstract

**Purpose:**

This study investigates a large‐area plane‐parallel ionization chamber (LAC) for measurements of dose‐area product in water (*DAP*_w_) in megavoltage (MV) photon fields.

**Methods:**

Uniformity of electrode separation of the LAC (PTW34070 Bragg Peak Chamber, sensitive volume diameter: 8.16 cm) was measured using high‐resolution microCT. Signal dependence on angle *α* of beam incidence for square 6 MV fields of side length *s =* 20 cm and 1 cm was measured in air. Polarity and recombination effects were characterized in 6, 10, and 18 MV photons fields. To assess the lateral setup tolerance, scanned LAC profiles of a 1 × 1 cm^2^ field were acquired. A 6 MV calibration coefficient, *N*_D__,w,_ _LAC_, was determined in a field collimated by a 5 cm diameter stereotactic cone with known *DAP*_w_. Additional calibrations in 10 × 10 cm^2^ fields at 6, 10, and 18 MV were performed.

**Results:**

Electrode separation is uniform and agrees with specifications. Volume‐averaging leads to a signal increase proportional to ~1/cos(*α*) in small fields. Correction factors for polarity and recombination range between 0.9986 to 0.9996 and 1.0007 to 1.0024, respectively. Off‐axis displacement by up to 0.5 cm did not change the measured signal in a 1 × 1 cm^2^ field. *N*_D__,w,_ _LAC_ was 163.7 mGy cm^−2^ nC ^−1^ and differs by +3.0% from the coefficient derived in the 10 × 10 cm^2^ 6 MV field. Response in 10 and 18 MV fields increased by 1.0% and 2.7% compared to 6 MV.

**Conclusions:**

The LAC requires only small correction factors for *DAP*_w_ measurements and shows little energy dependence. Lateral setup errors of 0.5 cm are tolerated in 1 × 1 cm^2^ fields, but beam incidence must be kept as close to normal as possible. Calibration in 10 × 10 fields is not recommended because of the LAC's over‐response. The accuracy of relative point‐dose measurements in the field's periphery is an important limiting factor for the accuracy of DAP_w_ measurements.

## 1 INTRODUCTION

The dosimetry of small megavoltage (MV) photon fields is difficult. The physical and theoretical challenges are outlined in several recent articles.^1^, ^2^, ^3^ These challenges have been recognized by major medical physics organizations and an IAEA and AAPM joint task group (TG 155) has been formed to provide guidance for medical physicists with respect to small field dosimetry. Meanwhile, a draft of the German Industry Standard DIN 6809‐8, detailing procedures for small field dosimetry, has become available for public comment.^4^ Overall, there is a considerable amount of research activity investigating the measurement and modelling of small photon fields, with two recent publications discussing those issues in detail.^5^, ^6^

Radiotherapy treatment planning systems require measured beam data, such as output ratios (ORs), percentage depth dose curves (PDDs), and tissue‐phantom‐ratios (TPRs). It is challenging to measure these data accurately for small photon fields, because of uncertainties in detector alignment with the field's axis as well as volume‐averaging effects as the field size approaches detector size. Lateral electronic disequilibrium and changes in energy spectrum of the beam with subsequent changes in detector response need also be considered.

### 1.A Dose‐area‐product and large area chambers

An alternative to central‐axis point‐dose measurements is the Dose‐Area‐Product (*DAP*). Detectors with sensitive volumes many times larger than the beam have the advantage of integrating dose with high precision independently of uncertainties in lateral alignment with the field axis. In practice, a Large‐Area plane‐parallel Chamber (LAC) has a response proportional to the absorbed Dose‐Area‐Product in water (*DAP*_w_). The accurate measurement of *DAP*_w_ allows a precise determination of on‐axis dose output of small photon fields, provided that the relative two‐dimensional dose distribution is accurately known from measurements with radiosensitive film or scanning detectors.

Several publications describe the use of a LAC, the PTW Bragg Peak chamber type 34070‐2.5 (PTW Freiburg, Germany), for measuring integral dose in small megavoltage photon fields.^7^, ^8^, ^9^, ^10^ Djouguela et al first described remarkable properties of *DAP*_w_ measurements of small fields, such as the similarity of depth‐dose curves at constant source‐to‐detector distance (*DAP*_w_‐TPR) and constant source‐to‐surface distance (*DAP*_w_‐PDD) and the invariance of those curves with field size.^7^ Sanchez‐Doblado et al derived OR of small fields by combining measurements of *DAP*_w_ and two‐dimensional dose distributions measured with radiochromic film, and found good agreement with diode measurements and Monte Carlo calculations.^8^ The Bragg Peak chamber has even found use as an upstream in‐line beam monitor by Lechner et al;^9^ and Heidorn et al have introduced the LAC as a quality assurance device in order to verify the constancy of the beam area for a Cyberknife Iris Variable Aperture Collimator.^10^

Since the PTW type 34070‐2.5 chamber has shown some potential useful applications for small field dosimetry measurements,^7^, ^8^ it was chosen as one of the detectors to measure *DAP*_w_ at the Australian Radiation Protection and Nuclear Safety Agency's (ARPANSA) linear accelerator, which is the reference source for dosimetric calibration and audit services provided by ARPANSA.

*DAP*_w_ is also a quantity of interest investigated by the MedExtRT project (http://radiotherapy-emrp.eu/), which aims to improve the dosimetry for small and composite MV photon beams. In this context, *DAP*_w_ has been investigated in the setting of various other primary standard laboratories, such as the National Physics Laboratory (NPL), UK, the Laboratoire Nationale Henry Becquerel in Paris, France, and the Istituto Nazionale di Metrologia delle Radiazioni Ionizzanti in Rome, Italy.^11^, ^12^, ^13^, ^14^, ^15^, ^16^, ^17^, ^18^

### 1.B Requirements for a suitable DAP chamber

We investigated whether the PTW type 34070‐2.5 chamber has the performance characteristics desirable of a reference *DAP*_w_ detector, by measuring signal reproducibility, linearity, long‐term stability, effects of recombination and polarity, angular sensitivity, and extra‐cameral effect. We used a microCT to determine whether the distance between the LAC's electrodes is uniform, and we measured the energy‐dependency of response in 6, 10, and 18 MV beams.

We calibrated the chamber in terms of *DAP*_w_ by cross‐calibration in a 6 MV photon field with known *DAP*_w_, and compared this to published values. We performed this calibration in fields that were either smaller or much larger than the LAC.

## 2 MATERIALS AND METHODS

### 2.A LAC performance as a reference detector

**Figure 1:**
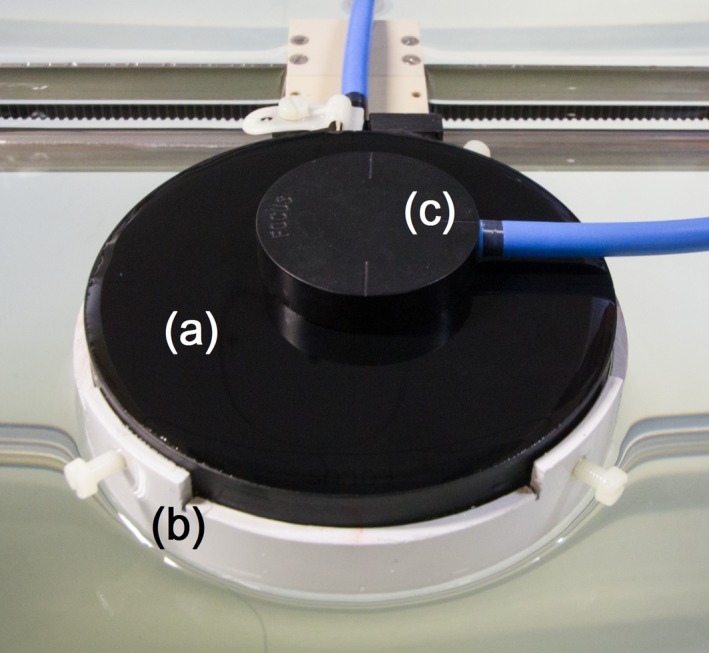
Photograph of the large‐area plane‐parallel ionization chamber (a) (outer diameter 10.4 cm), mounted with an in‐house holder (b). A small amount of water is present on top of the chamber. For size comparison, a PTW Roos chamber (c) is placed on top of the LAC.

The Bragg Peak chamber Type 34070‐2.5 (PTW Freiburg, Freiburg, Germany) is a waterproof, large‐area plane‐parallel ionization chamber (Fig. 1). The cylindrical lacquered PMMA body has an outer diameter of 103.95 mm and a height of 12.95 mm. The internal cylindrical air cavity is nominally 2.0 mm thick with a diameter of 84.0 mm. According to specifications, the entrance window has a physical thickness of 3.47 mm, comprised of 0.1 mm outer lacquer layer, 3.35 mm PMMA and the 0.02 mm graphite electrode, which in total corresponds to a water‐equivalent thickness of 0.4 g/cm^2^. The graphite collecting electrode is distal to the entrance window and has a diameter of 81.6 mm, hence the volume of air over which ionization is collected is 10.5 cm^3^ and its sensitive area is 52.25 cm^2^. The sensitive volume is guarded by a 1.1 mm wide guard ring and is vented to the atmosphere via a 1.5 m long waterproof cable. A schematic drawing of the chamber can be found in Djouguela et al.^7^ We used the center of the inner surface of the entrance window as the effective point of measurement (EPOM).

The chamber was irradiated with 6, 10, and 18 MV pulsed photon radiation generated by ARPANSA's linear accelerator (Elekta Synergy, Elekta AB, Stockholm, Sweden). This linac has main jaws in the in‐plane direction, and 1 cm wide Multi‐Leaf Collimators (MLC) with backup jaws in the cross‐plane direction.

A reference–class Unidos Webline electrometer (PTW Freiburg, Freiburg, Germany) was used to provide the polarizing voltage and collect the charge *M*_LAC_ produced in the LAC. An operating voltage of +400 V was used, with the collecting electrode at negative potential (CEN). This voltage is the maximum voltage recommended by the manufacturer. It results in minimal recombination losses while the LAC is operating in the ion‐chamber region. *M*_LAC_ was corrected for changes in air density by applying a correction factor *k*_TP_.^19^ The reference values for standard temperature and pressure are 20°C and 101.325 kPa, respectively. The so‐corrected charge is denoted as *M*_LAC,cor_.

#### 2.A.1 MicroCT analysis

The *DAP*_*w*_‐method requires a uniform dose‐response of the LAC across its sensitive area, and this in turn calls for a constant electrode separation. In order to measure electrode separation across the chamber area and also to assess details of the LAC's internal geometry, a high‐resolution computed tomography (CT) image of the LAC has been obtained (HR‐pQCT, XtremeCT II, SCANCO Medical AG, Brüttisellen, Switzerland). The spatial reconstruction accuracy was verified with a test object of known geometry (Phantom KP70, SCANCO Medical AG). X‐ray transmission signals (60 kVp) of the LAC were reconstructed to a 3D CT data set of 1274 images with voxels of 0.082 mm side length. All images were inspected visually for high‐density material.

The thicknesses of the chamber body and the internal air cavity were measured by performing an edge analysis of the CT data using an in‐house routine written in MATLAB^®^ (The MathWorks Inc., Natick, MA, USA). While keeping the resolution of the voxel space at 0.082 mm in the chamber's front‐to‐back direction, the data were resampled by averaging over 20 × 20 voxels (1.64 mm × 1.64 mm) along the chamber's longitudinal and transverse axes to reduce image noise and to increase edge detection accuracy. The resulting voxel columns along the chamber's front‐to‐back direction were analyzed for edges by finding the 50% values between air (voxel value = 0) and chamber body (average voxel value = 2200). The thickness of the chamber body was calculated from the average distance between the outer edges of all columns, and this value was compared to calliper measurements. The thickness of the internal air cavity was calculated from the average distance between the inner edges of all voxel columns within a 4.08 cm radius around the chamber center. The internal thickness of the air cavity was mapped and inspected visually for any systematic variations.

#### 2.A.2 Linearity and reproducibility

The reproducibility and linearity of response were determined in 6, 10, and 18 MV beams of 10 × 10 cm^2^ field size at 100 cm SSD and 10 cm depth. Reproducibility was calculated as the standard deviation of *M*_LAC,cor_ in 10 consecutive irradiations with 100 MU. Linearity was defined as the residual *R* ^2^ of the regression analysis of *M*_LAC,cor_ over MU, whereby the chamber was irradiated three times each with 25, 50, 100, 200, and 400 MU. An external transmission ionization chamber mounted on the shadow tray attached to the linac head was used to monitor and correct for variations in linac output (Type 7862, PTW Freiburg, Freiburg, Germany, diameter 96.5 mm, sensitive volume 17.6 cm^3^). We did not expect a change in the LAC's linear behaviour when reducing the field size below 10 × 10 cm^2^, and we did not perform a separate assessment of linearity in fields that are smaller than the LAC's sensitive diameter. The reproducibility of *M*_LAC,cor_ in the 5 cm diameter calibration field was expressed as the relative standard deviation of *M*_LAC,cor_.

#### 2.A.3 Long‐term response stability

Long‐term changes of response were assessed by exposing the LAC to a 20 MBq Strontium‐90 check source (PTW T48010, PTW Freiburg, Freiburg, Germany) at monthly intervals under fixed geometry. The LAC was placed on a 5 cm thick slab of Plastic Water^®^ (CIRS Inc., Norfolk, Virginia, USA) to provide uniform backscatter. A holder, constructed from poly‐vinyl chloride (PVC), suspended the source at a distance of 5 cm from the entrance window. *M*_LAC,cor_ was measured over 120 s and corrected for source decay.

#### 2.A.4 Saturation and polarity correction

The saturation correction factor *k*_s_ for the LAC was measured by changing the bias voltage *U* between +80 V and +400 V in 10 × 10, 4 × 4 and 1 × 1 cm^2^ 6 MV beams, at a depth in water of 1.5 cm and a SSD of 100 cm. Variations in linac output were accounted for with a monitor chamber (Farmer‐type, FC65‐G, IBA Dosimetry GmbH, Schwarzenbrueck, Germany), suspended laterally and upstream from the LAC within the 10 × 10 cm^2^ field. For the 1 × 1 and 4 × 4 cm^2^ field size, the monitor chamber was suspended in water 4 cm below the LAC on the beam central axis. The results were analyzed using a Jaffe plot. *M*_LAC,cor_(*U*)^−1^ was normalized to *M*_LAC,cor_(+400 V)^−1^ and plotted against *U* ^−1^. A linear regression analysis was performed to assess whether the LAC's nominal operating voltage of +400 V is appropriate. *k*_*s*_ was derived from the intercept of the fitted curves at *U* ^−1^ = 0. *k*_s_ was also measured using the two‐voltage technique and quadratic fit coefficients provided in TRS‐398, at voltage ratios *U*_1_:*U*_2_ of 1:2, 1:4, and 1:5, where *U*_2_ = +400 V.^19^

Supplementary values of *k*_*s*_ (voltage ratio *U*_1_:*U*_2_ = 1:4) have been determined for field sizes of 1 × 1, 3 × 3, 5 × 5, and 10 × 10 cm^2^ at SSD and depth combinations of 100 and 10 cm, 90 and 10 cm as well as 80 and 20 cm, without a monitor chamber. Under the same setup, the polarity correction factor *k*_*pol*_ was determined using the method described in TRS‐398.^19^

#### 2.A.5 Response anisotropy and extra‐cameral effect

The change of response with change of angle of beam incidence *α* was measured in air without build‐up. The linac was set to gantry angle *α* = 0° and a large 6 MV field (20 × 20 cm^2^) was selected. Using a large floor retort stand, the LAC was mounted with the entrance window toward the source, so that its EPOM was on the beam central axis and at the linac's gantry rotation isocenter, at 100 cm from the source. The chamber was irradiated with 100 MU at varying gantry angles *α* between 0° and 15°, *M*_LAC_ was recorded and normalized at *α* = 0°. The procedure was repeated with a 1 × 1 cm^2^ field.

The extra‐cameral effect was evaluated under broad beam conditions in a water phantom. The chamber was positioned at depth dose maximum (1.5 cm) in a 20 × 20 cm^2^ square 6 MV photon field. Approximately 1 m of the cable was suspended downstream and lateral to the chamber within the broad field and the signal *M*_*LAC*_ per MU with and without the cable in the beam was compared.

#### 2.A.6 LAC response vs lateral off‐axis displacement

Djouguela et al have found that the response of a PTW 34070 type chamber does not change with lateral off‐axis displacement of up to 2 cm in a 1 × 1 cm^2^ square field in water at 100 cm SSD.^7^ It is important to quantify the lateral setup tolerance of the LAC when measuring in fields smaller than the LAC's sensitive diameter, because this dictates the requirements for positioning accuracy of user's chosen combination of LAC and phantom. The LAC was scanned across a 1 × 1 cm^2^ field in the in‐plane and cross‐plane direction at 30 cm depth and at depth dose maximum. The center of the LAC was scanned from −8.0 cm to +8.0 cm. A customized holder for a scanning water tank (Wellhofer Blue Phantom, IBA Dosimetry GmbH, WLH = 50 cm × 50 cm × 40 cm) was constructed for this purpose (Fig. 1).

### 2.B Determination of LAC calibration coefficient

The aim of determining a calibration coefficient *N*_D,w,LAC_ for the LAC is the ability to perform measurements of *DAP*_w_ in small 6 MV photon beams. Under knowledge of *DAP*_w_, it is then possible to derive the output factor for a small field from the relative dose distribution, as demonstrated by Sanchez‐Doblado et al.^8^ A further use may be that of using the LAC as a transfer dosimeter to enable the calibration of small field detectors in a small reference field against a primary standard in primary standards laboratories, as outlined by Dufreneix et al.^15^

For clarity, the methods required for determining *N*_D,w,LAC_ are described first. Table 1 lists the experimental setups under which we determined *N*_D,w,LAC_.

**Table 1:** A list of experiments, their purpose, and corresponding field parameters. The SSD was 100 cm and the measurement depth was 10 g/cm^2^

Purpose(s)	Photon energy	Field collimation	Relative dose integral measurement
Determination of *N*_D,w,LAC_ in small field with known *DAP*_w_	6 MV	5 cm stereotactic cone	Film (EBT3) + Profiles (EFD, CC13)
Verification of *N*_D,w,LAC_ in small field with known *DAP*_w_	6 MV	4 × 4 cm^2^ jaw and MLC	Film (EBT3)
Dependency of LAC response on photon energy	6, 10 & 18 MV	10 × 10 cm^2^ jaw and MLC	Profiles (CC13)
Comparison of *N*_D,w,LAC_ determined in broad fields to (1)			

#### 2.B.1 General method of DAP_w_‐based LAC calibration

*M*_*LAC,cor*_ is related to *DAP*_w_ by multiplying with a calibration coefficient *N*_D,w,LAC_:

(1) $$DAP_{w}=M_{LAC,cor}\cdot N_{D,w,LAC}\cdot k_{i}$$

where *M*_*LAC,cor*_ is the charge collected by the LAC and corrected for air density; and *k*_*i*_ are correction factors for ion collection efficiency (*k*_s_), polarity effect (*k*_pol_), and electrometer collection efficiency (*k*_elec_).

The relationship between *DAP*_w_ and the central‐axis dose, *D*_*w,*CAX_, is given by

(2) $$DAP_{w}=\int \int^{A}D_{w}\left(r\right)dr=D_{w,\mathrm{CAX}}\cdot \int \int^{A}R\left(r\right)dr$$

where *R*(***r***) is the relative two‐dimensional dose distribution, normalized to unity at central axis (***r*** = 0), in the plane of the LAC detector. The double integral extends over *A*_LAC_, the LAC's sensitive area. $\int \int^{A}$ *R*(***r***)*d**r*** has been determined with radiochromic film and scanned dose profiles in the 5 cm diameter calibration field at 100 cm SSD and at 10 g/cm^2^ depth, as described in the following two sections.

#### 2.B.2 Relative dose integral – film method

EBT3 film (EBT3, Ashland Inc., Covington, Kentucky, USA) has been shown to exhibit minimal energy dependence in megavoltage photon radiation, but it under‐responds in kilovoltage x rays by about 5% to 15%.^16^, ^20^ This affects the accuracy of film dose measurements in the periphery of the field, where low‐energy scatter is more prevalent (see, for example, Chofor et al and by Kry et al).^21^, ^22^ Because complete information on the photon spectrum of the 5 cm diameter calibration field was not available at the time of calibration, we have determined an EBT3 calibration curve in a 10 × 10 cm^2^ field on central axis and subsequently made an allowance for the EBT3 under‐response in the periphery of the calibration field in our uncertainty budget. All film measurements were performed in a solid phantom (Plastic Water^®^– The Original, CIRS Inc., Norfolk, Virginia, USA).

Because the response of EBT3 can vary within a sheet, as shown by Micke et al, a calibration curve based on net‐optical density (Δ*OD*) was created.^23^ Films were scanned immediately prior to exposure and 24 hr after exposure using a flatbed transmission scanner (Epson Perfection V700, SEIKO Epson Corporation, Suwa, Japan) at 300 dpi. The red channel pixel values *PV*_red_ were smoothed using a 30 × 30 pixel median filter, to remove the influence of dust and foreign particles. To investigate the effect of filtering, the complete analysis was repeated without any filtering. *PV*_red_ were converted to optical densities (*OD*) using the formula

(3) $$OD=-{\text{log}}_{10}\left(PV_{\mathrm{red}}/2^{16}\right)$$

Pre‐ and postexposure images were spatially coregistered using marks on the film. The preexposure *OD* was subtracted from the postexposure *OD* to obtain Δ*OD*, the net‐optical density. Δ*OD* was converted to dose (unit: Gy) using the formula

(4) $$\begin{array}{cc} D_{w} & =a_{o}+a_{1}\cdot \Delta OD+a_{2}\cdot \Delta OD^{2}+a_{3}\cdot \Delta OD^{3}+a_{4}\cdot \Delta OD^{4}\\ & \;+a_{5}\cdot \Delta OD^{5} \end{array}$$

where *a*_0_ = 0, *a*_1_ = 5.29081, *a*_2_ = 62.94971, *a*_3_ = −295.56757, *a*_4_ = 908.96498, *a*_5_ = −981.35406. The coefficients *a*_i_ are based on a 5^th^ order polynomial fit to the *D*_w_ vs Δ*OD* curve ranging from 0 to 2.8 Gy, which has been obtained by cross‐calibration against a reference ionization chamber.

To improve the accuracy of the film dosimetry in the low‐dose region, we employed a method similar to that presented by Sanchez‐Doblado et al.^8^ Two pieces of film were exposed at a high (4000 MU) and at a low (400 MU) setting, to ensure that the out‐of‐field and in‐field dose were within the dynamic range of EBT3 in one of the exposures. To combine the two exposures, the dose map obtained at 4000 MU was divided by 10, and both dose maps were aligned to each other using their respective beam centers. The final dose map was created by choosing the higher of the two values from the two dose maps at any point, yielding a two‐dimensional dose distribution *D*_*w*_(*x,y*)*,* which was then normalized to central axis to obtain a relative dose distribution *R*(*x,y*).

##### 2.B.3 Uncertainty of the film dose measurement

**Figure 2:**
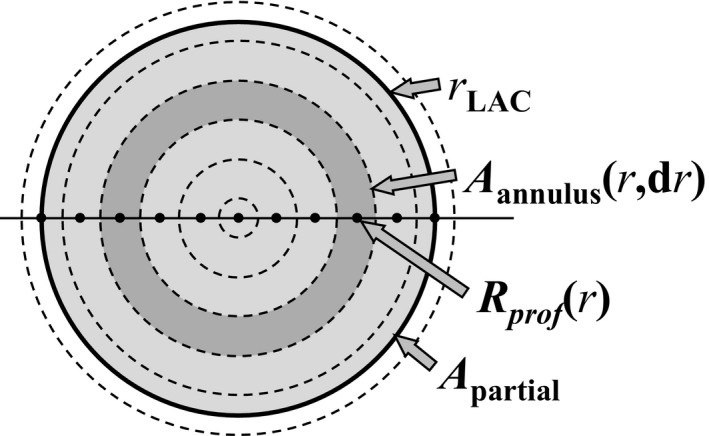
Illustration of area‐weighted dose averaging based on transverse relative dose profiles *R*_prof_(*r*) of a circular symmetric dose distribution. Abbreviations: *r*_LAC_: radius of large‐area chamber, *A*_annulus_(*r*,d*r*): area of annulus at off‐axis distance *r* and annulus width d*r*,* R*_prof_(*r*): off‐axis relative dose ratio at *r*,* A*_partial_: area of partial annulus, where the outer radius of the annulus is equal to *r*_LAC_.

For the film response within the geometric field boundaries, we have assumed a value of 2% (k = 1) for the local response of the EBT3 film, which is achievable using stringent film dosimetry protocols based on net‐optical density. In the area outside the geometric field boundary but still inside the LAC's sensitive diameter, we have added an extra 5% uncertainty to account for a potential under‐response of the EBT3 film. In that area, the mean photon energy is approximately 0.5 MeV, based on Monte Carlo data for a comparable setup (5 × 5 cm^2^ 6 MV field, 100 cm SSD, 10 cm depth) depicted in Fig. 2(c) in the publication by Chofor et al.^24^ Approximating from data presented by Dufreneix et al, the EBT3 response is reduced by a factor of 0.95 to 1.0 compared to 6 MV, and we have assumed 0.95 as the worst case.^16^ Hence, a 5% uncertainty term was added to the out‐of‐field EBT3 response uncertainty, increasing it to 7.0% (k = 1).

The relative uncertainty of ∫∫^*A*^ *R*(***r***)*d**r*** was calculated as the sum of the in‐field and out‐of‐field uncertainty, weighted by the relative contribution to the total integral.

#### 2.B.3 Relative dose integral – profile method

If the dose distribution across the circular integration area has a circular symmetry, then an alternative integration method using high‐resolution scanned dose profiles can be employed.^15^, ^25^, ^26^ Table 1 lists the type of detectors used for each instance where we used this method, a general description of which is given here. In order to obtain the relative dose integral, a small‐volume detector is scanned in water at the calibration depth in a star pattern across the beam. The dose profiles *R*_prof_(*r*) must intersect with and be normalized to the beam's central axis, where *r* = 0 cm. With *R*_prof_(*r*) and annuli of area *A*_annulus_(*r,*d*r*) as defined in Fig. 2, the relative dose integral over a circle with radius *r*_*LAC*_ is calculated from each half‐profile as the annular area‐weighted average dose multiplied by *π r*_*LAC*_^2^, thus

(5) $$\begin{array}{c} \int \int^{A_{\mathrm{LAC}}}R\left(r\right)dr=\pi r_{LAC}^{2}\cdot \frac{\sum_{r}\left[R_{prof}\left(r\right)\cdot A_{annulus}\left(r,dr\right)\right]}{\sum_{r}\left[A_{annulus}\left(r,dr\right)\right]} \end{array}$$

The thickness, *dr*, of the annulus is determined from the sum of the halfway distances to the two adjacent measurement points in the profile. The results from several transverse scans in a star pattern may be combined to improve the accuracy of this method and account for asymmetries in the beam profile.

∫∫^*A*LAC^ *R*(***r***)*d**r*** was measured for the 5 cm circular calibration field with two small detectors: a miniature thimble ionization chamber with a detector volume of 0.13 cm^3^ and operated at +300 V (CC13), and an unshielded electron diode (EFD, IBA Dosimetry GmbH). Detector choice was based on availability, well‐known properties, stability and adequate size for the purpose, as well as to provide two independent data sets. Each detector was scanned in the in‐plane, cross‐plane and in both diagonal directions.

#### 2.B.4 Determination of calibration coefficients in a field smaller than the LAC's sensitive area

The calibration coefficient for the LAC, *N*_D,w,LAC_, was determined in a 6 MV calibration field, for which *DAP*_w_ was previously measured. The field has a diameter of 5.0 cm at 100 cm SSD and is collimated with a stereotactic cone (Stereotactic Collimator, Elekta AB, Stockholm, Sweden, material: low‐melting‐point alloy). Cone collimation was chosen because of the greater reproducibility of the beam area from day to day compared to collimation be MLC and jaws alone. For example, ARPANSA's Elekta Synergy linac displays the values for jaw and MLC positions with a precision of 0.01 cm, but also tolerates submillimeter differences between the displayed and the desired jaw position. Assuming that the actual jaw/MLC position is within ±0.02 cm of the desired position in 95% of cases, then a field of side length *s* = 4.00 cm has a field area *A* of 16.0 cm^2^ where the area *A* has a relative uncertainty of 0.7% (k = 1). This significant source of uncertainty can only be avoided by using a fixed collimator.

Another reason for choosing a 5 cm diameter field for calibration instead of a broad field is the changes in photon fluence spectrum that occur as the field size is reduced. Because the intention is to use the LAC for small field dosimetry in the future, large differences in spectrum between the calibration field and the subsequent measurement conditions should be avoided.

All calibration measurements were performed at 100.0 cm SSD and at a depth of 10 g/cm^2^ in water.

The central‐axis dose *D*_*w,*CAX_ of the calibration field was determined with ARPANSA's secondary standard ionization chamber (NE2571 Farmer, Nuclear Enterprises Ltd., Sighthill, Scotland). The calibration coefficients for the NE2571 chamber were previously derived with an uncertainty of 0.5% (at k = 1 level) by direct comparison against the Australian primary standard graphite calorimeter in a 10 × 10 cm^2^ field for 6, 10, and 18 MV.^27^

Because the cavity of the NE2571 chamber has a length of 2.4 cm, a volume‐averaging effect in the 5 cm diameter 6 MV calibration field can occur due to a nonflat dose profile. A volume‐averaging correction factor was obtained from the available dose profile data obtained with the EBT3, the EFD, and the CC13 by averaging over the central 2.4 cm of the dose profile in the direction of the NE2571's longitudinal axis. We also assessed the effect of volume‐averaging by comparing output ratios (OR) measured with the NE2571 against those measured with a small volume ion chamber (CC13) for field sizes of 4 × 4 and 5 × 5 cm^2^ at 100 cm SSD and at 10.0 g/cm^2^ depth.

From Monte Carlo studies published by Chofor et al it is known that due to the NE2571's very small energy dependence, its response need only be corrected by a factor of 0.999 when reducing the size of a 6 MV field from 10 × 10 cm^2^ to 4 × 4 cm^2^.^24^, ^28^ Because in our case the calculated spectra for our 5 cm diameter calibration field were not yet available, we have not applied a correction factor for energy dependence and instead have added a relative uncertainty term of 0.2%.

We repeated the LAC calibration procedure in a 4 × 4 cm^2^ 6 MV field (without a stereotactic cone) to highlight differences in the resulting total uncertainty. The setup was identical to the one described above, but, due to the noncircular field shape, ∫∫^*A*LAC^ *R*(***r***)*d**r*** was derived from EBT3 only.

#### 2.B.5 Determination of calibration coefficients in a broad field

For the purpose of comparison to the calibration in the 5 cm diameter field and to understand the energy dependence of the LAC's response, *N*_D,w,LAC_ was determined for 6, 10, and 18 MV in a broad (10 × 10 cm^2^) field. The choice of broad field to investigate the LAC's energy dependence was dictated by the fact that the 5 cm stereotactic cone could not be used for energies above 6 MV, and the assumed large uncertainty associated with the beam area of the 4 × 4 cm^2^ field. Here, ∫∫^*A*LAC^ *R*(***r***)*d**r*** were calculated from four beam profile scans (in‐plane, cross‐plane, and diagonals) with a small‐volume ion chamber (CC13), while *D*_CAX_ was determined with the NE2571. Linac output variations were removed by using an external transmission monitor chamber (PTW Type 7862).

## 3 RESULTS

### 3.A Performance as a reference detector

#### 3.A.1 MicroCT analysis

**Figure 3:**
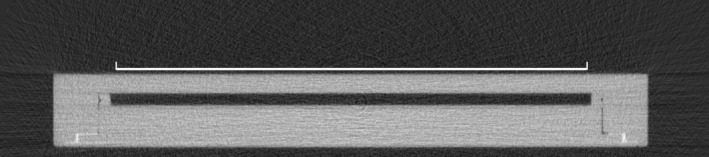
MicroCT image showing a central cross‐section of the LAC with the entrance window facing upwards. The length of the white line is 81.60 mm, corresponding to the nominal diameter of the sensitive collecting volume.

Figure 3 shows a microCT cross‐section of the LAC. The chamber body is of uniform density, with only a very small amount of high‐density material downstream and to the side of the sensitive volume.

The microCT scanner pixel scaling accuracy was confirmed to within 0.2% using the test object. The external thickness of the LAC was measured as 12.75 ± 0.03 mm (mean ± 1 SD), which agrees with calliper measurements of 12.78 ± 0.01 mm, but is less than the nominally stated 12.95 ± 0.15 mm. The height of the internal air cavity was determined from a total of 2012 measurements points as 2.01 ± 0.03 mm, which agrees with the manufacturer's stated value of 2.0 ± 0.06 mm. There is no evidence of any spatial variation in electrode separation across the whole chamber volume.

Significant high‐density components were only visible where the cable enters the chamber via a short tubular aluminium stem. A small cavity (approximately 0.15 cm^3^) was visible adjacent to the stem where the individual cables exit the waterproof cable within the chamber body.

#### 3.A.2 Linearity and reproducibility

The reproducibility of *M*_LAC_ was better than 0.04% in the broad field and 0.07% in the 5 cm diameter calibration field, respectively. The linearity for absorbed doses between 0.5 and 4 Gy, measured as the residual square error of a linear regression analysis, was *R* ^2^ = 1.0000 for 6, 10, and 18 MV photon beams. Maximum and average relative deviation of *M*_LAC_ from linearity were 0.17% and <0.1%, respectively.

#### 3.A.3 Long‐term response stability

The reproducibility of setup with the check source was 0.4% (1 SD). Rotating the source resulted in changes <0.02%. The variation in chamber response to the check source over 9.5 months was within a range of 0.6%.

#### 3.A.4 Saturation and polarity correction

**Figure 4:**
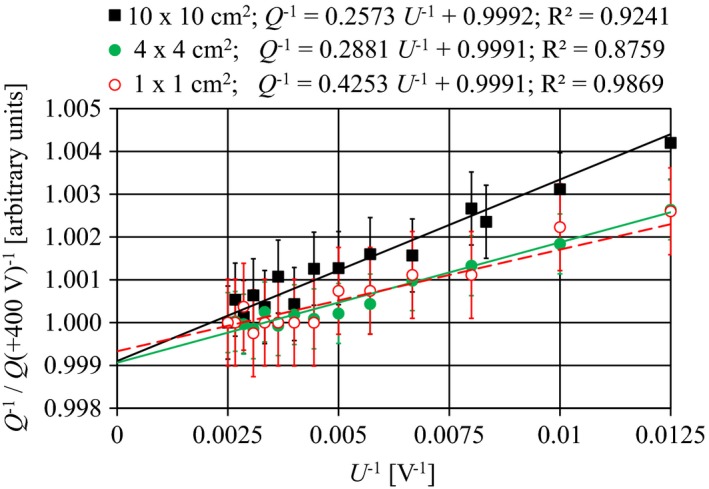
Jaffe plot normalized to *Q*(+400 V)^−1^ for field sizes of 10 × 10, 4 × 4, and 1 × 1 cm^2^ at 100 cm SSD. Least square fit of linear curves from +80 V to +400 V; the linear curve equations are shown in the figure. Error bars correspond to 1 SD. The LAC was irradiated with 6 MV at 1.5 g/cm^2^ depth in a water phantom at 100 cm SSD.

Jaffe plots are shown in Fig. 4. The intercept with the 1/Q axis lies at 0.9991 ± 0.0001, which corresponds to a value of *k*_s_ = 0.9991^−1^ = 1.0009 for 6 MV (Table 2). Further values for *k*_s_ determined with the two‐voltage method at a number of different field sizes and depths in water are listed in Table 3.

**Table 2:** Saturation correction factors *k*_s_ for 6 MV fields of different sizes. These were measured with the LAC at nominal operating voltage of +400 V and at 1.5 g/cm^2^ depth in a water phantom at 100 cm SSD

Side length of square field *s* (cm)	10	4	1
*k*_s_ determined from intercept of linear fit	1.0009	1.0008	1.0009
*k*_s_ determined from M(+400V)/M(+200V) (voltage ratio 1:2)	1.0012	1.0002	1.0007
*k*_s_ determined from M(+400V)/M(+100V) (voltage ratio 1:4)	1.0010	1.0006	1.0007
*k*_s_ determined from M(+400V)/M(+80V) (voltage ratio 1:5)	1.0020	1.0016	1.0016

**Table 3:** *k*_s_ and *k*_pol_ measured in broad and small square fields of side length *s* at different combinations of SSD, depth and beam energy. *k*_s_ was determined with the two‐voltage method using bias voltages of +400 V and +100 V

SSD, depth (cm)	Field side length *s* (cm)	6 MV		10 MV		18 MV	
		k_s_	*k*_pol_	k_s_	*k*_pol_	k_s_	*k*_pol_
100, 10	10	1.0007	0.9995	1.0014	0.9992	1.0018	0.9993
90, 10	10	1.0016	0.9986	1.0020	0.9988	1.0024	0.9989
	5	1.0012	0.9990	1.0016	0.9991	1.0018	0.9993
	3	1.0013	0.9990	1.0014	0.9992	1.0016	0.9994
	1	1.0010	0.9989	1.0012	0.9991	1.0012	0.9994
80, 20	10	1.0017	0.9986	1.0021	0.9987	1.0024	0.9988
	5	1.0007	0.9994	1.0012	0.9993	1.0015	0.9995
	3	1.0013	0.9988	1.0013	0.9991	1.0014	0.9993
	1	1.0009	0.9989	1.0010	0.9994	1.0011	0.9994
Uncertainty (1 SD)		±0.0005	±0.0005	±0.0005	±0.0005	±0.0005	±0.0005

Values for *k*_pol_ are within 0.05% of 0.9990 for an operating voltage of +400 V (CEN), as shown in Table 3.

#### 3.A.5 Response anisotropy and extra‐cameral signal

**Figure 5:**
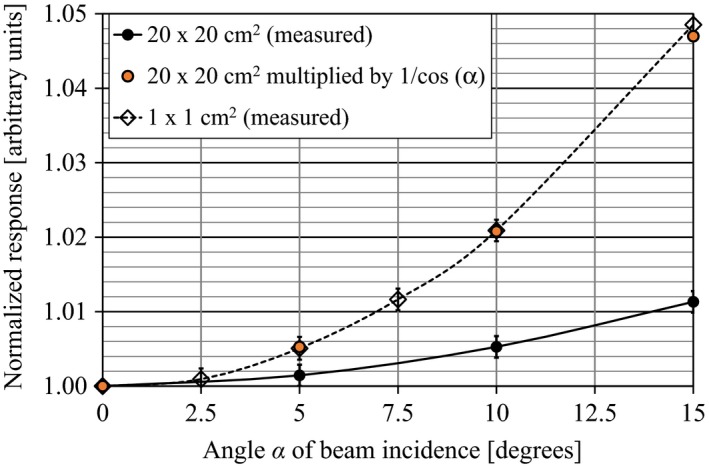
Normalized in‐air response vs angle of beam incidence *α* for a broad (20 × 20 cm^2^) and a small (1 × 1 cm^2^) square 6 MV photon fields, measured at 100 cm SAD to the LAC's effective point of measurement. The response is normalized at *α* = 0°. The dashed line was calculated by multiplying the normalized response in the broad field by a factor of 1/cos(*α*).

The LAC's response in the 6 MV broad beam increases slowly with *α* at a rate of 0.25% per 5° (Fig. 5). For the small 1 × 1 cm^2^ 6 MV field, a very significant additional increase in response is evident. An explanation for this effect, which was not investigated in other MV photon beam energies, is given in the discussion and the thus predicted response anisotropy is shown in the figure.

The extra‐cameral effect was not detected, as its influence was less than the reproducibility of the LAC's response (0.1%).

#### 3.A.6 LAC response vs lateral off‐axis displacement

**Figure 6:**
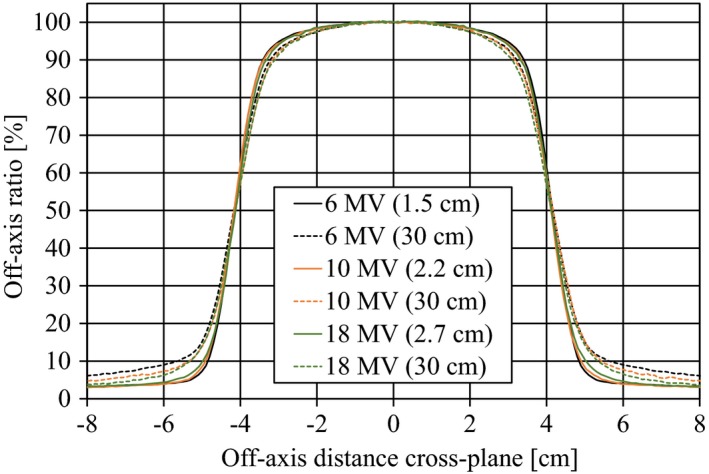
Cross‐plane off‐axis ratios measured with the LAC in a 1 × 1 cm^2^ field at depth of dose maximum and at 30 g/cm^2^ depth in a water phantom positioned at 100 cm SSD. The depth of dose maximum for 6, 10, and 18 MV is 1.5, 2.2, and 2.7 g/cm^2^, respectively. The center of the LAC was scanned from −8.0 to +8.0 cm.

The flat region in the center of the lateral response of the LAC in a 1 × 1 cm^2^ beam shows that there is less than 0.2% variation of *M*_*LAC*_ with lateral displacement of up to 0.5 cm (Fig. 6). At depth of *d*_max_, the out‐of‐field LAC signal is approximately equal to 4% of the central‐axis dose, and at the deeper depth of 30 g/cm^2^ this value has risen to 8% and 6% for 6 and 10 MV, respectively.

### 3.B Determination of LAC calibration coefficient

All quantities used to determine the dose‐area calibration coefficient *N*_D,w,LAC_ and their corresponding uncertainties are summarized in Tables 4 and 5.

**Table 4:** Quantities and their values used in the determination of the dose‐area calibration coefficient *N*_D,w,LAC_ obtained in different fields. The LAC was irradiated in a water phantom at 100 cm SSD with the effective point of measurement at a depth of 10 g/cm^2^

Photon energyField collimation		6 MV	6 MV	6 MV	10 MV	18 MV
Quantity	Unit	5 cm cone	4 × 4 cm^2^	10 × 10 cm^2^	10 × 10 cm^2^	10 × 10 cm^2^
Determination of dose‐area‐product		Value				
*D*_w,CAX,NE2571_	mGy MU^−1^	7.379	7.159	7.998	8.080	8.073
∫∫^*A*LAC^ *R*(***r***)*d**r***	cm^2^	24.96	20.29	52.01	52.51	51.97
* DAP*_w_	mGy cm^−2^ MU^−1^	184.5	145.2	416.0	424.3	419.6
Determination of LAC response						
Monitor units	MU	100	100	200	200	200
M_LAC,cor_	nC	112.8	88.97	523.6	539.0	541.8
k_s_		1.001	1.001	1.001	1.001	1.002
*k*_pol_		0.999	0.999	0.999	0.999	0.999
*k*_elec_		1.000	1.000	1.000	1.000	1.000
***N***_**D,w,LAC**_	**mGy cm** ^−**2**^ **nC** ^**−1**^	**163.7**	**163.3**	**158.9**	**157.3**	**154.7**
Rel. Unc. (k = 1)		1.3%	2.6%	0.75%	0.75%	0.75%

The underline signifies that the value in this row is a product of the two preceding rows. It is an intermediate result.

The bold signifies that this is the final result. I intended to draw the readers attention to that row foremost.

**Table 5:** Summary of sources of uncertainties and their values

Photon energy	Relative standard uncertainty k = 1(%)		
	6 MV	6 MV	6, 10, 18 MV
Field collimation	5 cm cone	4 × 4 cm^2^	10 × 10 cm^2^
Source of uncertainty			
Determination of dose‐area‐product			
Central‐axis dose *D*_w,CAX,NE2571_	0.6	0.6	0.5
Relative dose integral ∫∫^*A*LAC^ *R*(***r***)*d**r***	1.0	2.4	0.25
Change of field size from 10 × 10 cm reference field	0.2	0.2	–
Dose‐Area‐Product *DAP*_w_	1.2	2.5	0.56
Determination of LAC response			
M_LAC,cor_	0.07	0.07	0.07
k_s_	0.1	0.1	0.1
*k*_pol_	0.05	0.05	0.05
*k*_elec_	0.05	0.05	0.05
Additional sources of uncertainty			
Reproducibility of linac output	0.2	0.2	–
Lateral misalignment (1 mm)	0.05	0.05	0.05
Misalignment at depth (1 mm)	0.4	0.4	0.4
Manufacturer's tolerance of radius of LAC's sensitive volume (0.1 mm)	0.25	0.25	0.25
Reproducibility of 4 × 4 cm^2^ field area	–	0.7	–
Combined uncertainties			
**Calibration coefficient ** ***N***_**D,w,LAC**_	**1.3**	**2.6**	**0.75**

The underline signifies that the value in this row is a product of the two preceding rows. It is an intermediate result.

The bold signifies that this is the final result. I intended to draw the readers attention to that row foremost.

#### 3.B.1 Experimental determination of the relative dose integral

**Figure 7:**
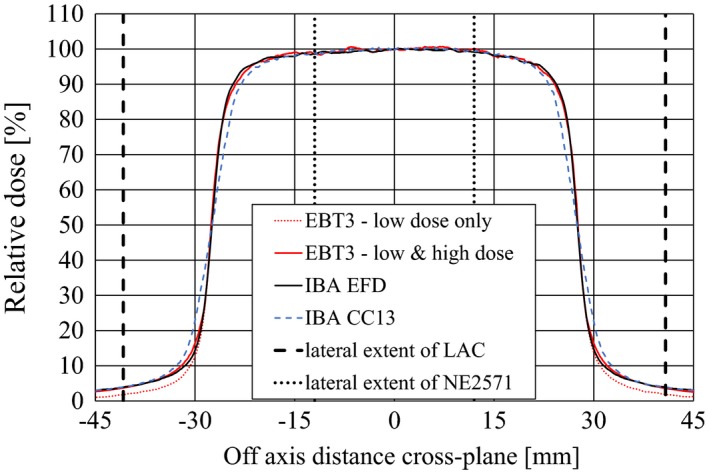
Cross‐plane relative dose profiles of a 6 MV beam at 10 cm depth and at 100 cm SSD, collimated by a 5 cm diameter stereotactic cone. The detectors and phantoms used are: EBT3 low‐dose exposure (400 MU, red dotted line) and a combination of low‐dose and high‐dose (4000 MU) EBT3 film exposures (red line) positioned perpendicular to central axis in a Plastic Water^®^ phantom, an IBA EFD unshielded electron diode (black line) and an IBA CC13 miniature ionization chamber (blue dashed line) positioned in a scanning water phantom. The lateral extent of the LAC's and the NE2571's sensitive volumes are indicated as vertical black dashed and dotted lines, respectively.

Figure 7 shows cross‐plane dose profiles of the 5 cm cone measured with the EBT3 low MU exposure, the combination of EBT3 low and high MU exposure, the CC13 and the EFD. Compared to the EFD, the EBT3 film shows slight penumbra broadening due to the 30 × 30 pixel median filter. Profiles obtained with the CC13 show a broadening of the penumbra due to the size of its sensitive volume. Most notable, however, is the under‐response of the low MU exposure EBT3 film in the low‐dose region of less than 20% of the central‐axis dose, particularly at relative doses below 10%, where the EBT3 measurement is approximately one half of that measured with the CC13 and the diode. In contrast, cross‐plane profiles based on the combination of high MU and low MU EBT3 exposure agree with the diode measurements to within 2% in the central region and outside the penumbra. In‐plane profiles display a similar behaviour of the low MU exposure EBT3 film (data not shown).

The relative dose integral ∫∫^*A*LAC^ *R*(***r***)*d**r*** evaluated to 25.17 ± 0.60 cm^2^ for the two‐film approach (1 SD = 2.6%). The film data outside the field boundary with the higher uncertainty of 7% contributed only 10% to the total dose integral. Using profiles measured with the small‐volume ionization chamber (CC13) and the unshielded diode (EFD), the integral was 24.83 ± 0.25 cm^2^ and 24.87 ± 0.25 cm^2^, respectively. All values agreed to within the Type A measurement uncertainties. The final value was the average of 24.96 ± 0.25 cm^2^ (1 SD = 1.0%).

Repeating the film analysis without the median filter resulted in a relative dose integral that was 0.2% less than with the 30 × 30 pixel median filter, and this difference is small compared to the overall uncertainty of the experiment.

Repeated calculations of ∫∫^*A*LAC^ *R*(***r***)*d**r*** from film data while shifting the center of the integral laterally, resulted in a less than 0.05% change per 1 mm of lateral displacement of the integral for distances of up to 3 mm in the in‐plane or cross‐plane direction.

#### 3.B.2 Determination of calibration coefficient in a field smaller than the LAC's sensitive area

The volume‐averaging effect of the NE2571 leads to a small under‐response in the 5 cm diameter calibration field. The associated correction factor was determined from dose profiles measured with the EBT3, EFD, and CC13 as 1.0011, 1.0051, and 1.0032, respectively. The arithmetic mean value of 1.003 ± 0.002 (1 SD) was chosen as the volume‐averaging correction factor. Application of this factor to the output ratio measured with the NE2571 in 5 × 5 cm^2^ field (OR_NE2571_ = 0.899) yields a value of 0.902, which was equal to the output ratio measured with a CC13 chamber (OR_CC13_ = 0.902).

The *DAP*_w_ of the calibration field was obtained following eq. (2) by multiplying the average relative dose integral measured with EBT3, EFD, and CC13 with the absolute central‐axis dose measured with the NE2571. Using eq. (1), subsequent division of *DAP*_w_ by (*M*_*LAC*,cor_ · *k*_*i*_) yielded *N*_D,w,LAC_ = 163.7 mGy cm^−2^ nC^−1^. The total standard uncertainty for this value is 1.3% (Tables 4 and 5). The stated uncertainty accounts for a possible deviation of *r*_LAC_ from the manufacturer's specification by 0.1 mm.

Repeating the calibration procedure in a square 4 × 4 cm^2^ MLC field, we obtained a calibration coefficient of 163.3 mGy cm^−2^ nC^−1^, which differs from the first value by −0.25%. The second result has a larger uncertainty of 2.6% based on the assumption that the MLC and jaw positions are reproducible only to ±0.02 cm and that the relative dose integral for this field was 20.29 cm^2^ from film measurements alone (1 SD = 2.4%). The volume‐averaging correction was determined as 1.0035 ± 0.002 from the film profile and the comparison of OR measured with the NE2571 and the CC13.

#### 3.B.3 Determination of calibration coefficients in a broad field

*N*_D,w,LAC_ determined in the 10 × 10 cm^2^ field were 158.9, 157.3, and 154.7 cGy cm^−2^ nC^−1^ (1 SD = 0.75%) for 6, 10, and 18 MV, respectively, (see also Tables 4 and 5). The relative dose integrals used for these results were derived from dose profiles obtained with the CC13 only.

## 4 DISCUSSION

Uniform thickness of the sensitive volume is a requirement for correct planar dose‐integration, as even a change of as little as 0.01 mm will change the volume of air (and hence the signal) by 0.5% in a 2 mm wide air cavity. By analyzing microCT data, we were able to verify the vendor‐specified data and to visually inspect for any systematic variations, which were not found. High‐resolution microCT was also an appropriate tool to scan for high‐density material inside the chamber and revealed the presence of a small air cavity near the stem, a feature that is not seen on the drawings in the LAC's manual.

Our lateral profile measurements produced a flat response with off‐axis positions of a few mm in a 1 × 1 cm^2^ 6 MV beam. The tolerance is about 0.5 cm, which is less than quoted by Douguela et al.^7^ We were somewhat surprised by the relative magnitude the out‐of‐field signal, which is approximately 4% of the central‐axis signal at *d*_max_ for all three photon energies and presumably arises due to scatter and leakage. Even for a 1 × 1 cm^2^ beam, the 8 cm diameter LAC is not sufficient to encompass all of the beam. Caution is recommended in any *DAP*_w_ analysis that assumes the entire beam is measured, or that the value of the dose at the edge of the detector is zero.

For primary beams that fit entirely within the LAC's sensitive area, the increase in signal *M*_LAC_ with angle of incidence *α* can be explained by the geometric relationship between *α* and the volume of air within the LAC that is inside the field borders. In a first approximation for a narrow parallel beam of side length *s* < *r*_LAC_, incident with angle *α*, the irradiated sensitive volume changes by a factor equal to ~1/cos(*α*). To approximate the measured change in ionization, this volume‐averaging effect was multiplied by the anisotropic response measured in the broad beam. The approximation matches closely to the experimental results for a 1 × 1 cm^2^ beam (see Fig. 5). These results highlight the crucial dependence of *M*_*LAC*_ on the mass of air in which ionization occurs. An incidence angle of *α* = 2.5° would cause a 0.1% increase in signal for fields smaller than the LAC's sensitive area. In cases where the LAC is positioned with its entrance window facing toward a photon source at 100 cm distance, any photon beam originating from the source will intersect with the LAC at an incident angle of *α* < 2.5°, and the increase in signal due to beam incidence would be less than 0.1%.

Ion recombination losses are generally very small. The linear slope of Q^−1^ vs U^−1^ at operating voltages between +100 V ≤ *U* ≤ +400 V for 6 MV indicates that the LAC operates in the ion‐chamber region and that the two‐voltage method can be used to derive *k*_s_ if *U*_2_ > 100 V (Fig. 4). The associated correction factor *k*_s_ increases slightly with beam energy and with field size. The latter effect can be explained by the higher dose rate in larger fields, whereby an increased charge‐density within the LAC's sensitive air volume leads to an increase in general recombination. Because the values for *k*_s_ are generally so close to unity, it is appropriate to use a *k*_s_ value determined in a 5 × 5 cm^2^ field and apply this value to any field size down to 1 × 1 cm^2^ without compromising measurement accuracy.

Measurements of the relative dose integral with EBT3 film were consistent with measurements using scanned profiles, as long as the two‐film method was used to obtain in‐field and out‐of‐field dose. Applying a 30 × 30 pixel median filter during the film analysis did not significantly alter the calculated dose integral compared to no filter, despite an apparent penumbra broadening. There was very good agreement of the calculated relative dose integral for the small ionization chamber (CC13) and the unshielded diode (EFD), despite their different sizes in sensitive volume. This is because the numerical integration process removes the effect of any blurring due to detector size, as long as the integration area is much larger than the size of the small detector.

From an analysis of the dose map obtained with film, it is evident that the setup alignment of the LAC in the calibration beam does not require a lateral displacement accuracy of better than 2 mm without loss of calibration accuracy.

The calibration values *N*_D,w,LAC_ derived here in a 5 cm diameter field are consistent with those determined in a 4 × 4 cm^2^ MLC field. However, in a 10 × 10 cm^2^ square MLC field, the coefficient is reduced by 3.0%, which indicates an over‐response of the LAC. The increased response of the chamber may be attributed to an additional ionization caused by secondary electrons scattering laterally into the sensitive volume after crossing the 1.1 mm wide guard ring, which is not wide enough to effectively screen those electrons. There is also a remote possibility that ionization of air within the small cavity near the stem contributes to the measured charge.

The calibration coefficient measured in the broad 6 MV field (158.9 mGy cm^−2^ C^−1^) differs by 5.6% from the values reported by Djouguela et al for this chamber type.^7^ There, the authors present a coefficient of (1.730 ± 0.002) × 10^8^ Gy cm^−2^ C^−1^ for 6 MV at 995 hPa and 23°C. Derivation of the calibration coefficient for reference environmental conditions of 20°C and 1013.25 hPa yields a value of 168.0 mGy cm^−2^ C^−1^ (1 SD = 0.1%). As a different chamber was used and manufacturing tolerances for the sensitive air volume allow a range of up to 7%, the difference compared to the here found calibration value is within an expected range.

Compared to the response in a 6 MV beam, we noted an increase in the LAC's response of 1.0% and 2.7% in 10 and 18 MV, respectively. Djouguela et al observed a broad‐field calibration value for 15 MV of (1.700 ± 0.002) × 10^8^ Gy cm^−2^ C^−1^ at 995 hPa and 23°C, and this marks an increase in response of 1.7% compared to their 6 MV beam, which is consistent with the trend seen in our values.^7^ This increase can largely be explained by the changes in the restricted Spencer‐Attix water/air stopping power ratio, *s*_w,air_. Following TRS‐398, the known TPR_20,10_ values for ARPANSA's MV photon beams can be used to calculate a *s*_w,air_ as 1.120 for 6 MV (TPR_20,10_ = 0.673), 1.105 for 10 MV (TPR_20,10_ = 0.734), and 1.089 for 18 MV (TPR_20,10_ = 0.777).^19^, ^29^ The expected change in response for the LAC when going from 6 to 10 and to 18 MV is +1.3% and +2.8%, and this agrees reasonably with the actual observed increase of 1.0% and 2.7%.

Dufreneix et al have given 6 MV calibration coefficients for a plane‐parallel ionization chamber with a plate separation of 2.0 mm and a diameter of 3 cm.^15^ The values obtained via cross‐calibration against a calorimeter in circular fields of diameter 2.0, 1.0, and 0.75 cm diameter were quoted as 158.56, 155.11, and 155.25 mGy cm^−2^ nC^−1^ at standard temperature and pressure. These values have an uncertainty of <0.9%, are within 3.2% of those presented in this paper, which is remarkable given the only common property of the two chamber types is their nominal plate separation of 2.0 mm.

## 5 CONCLUSIONS

A large‐area plane‐parallel ionization chamber (LAC) with a sensitive area of 8.16 cm diameter was investigated for MV photon beam dosimetry. A calibration coefficient *N*_D,w,LAC_ based on dose‐area product in water (*DAP*_w_) was determined in a circular field of 5 cm diameter and in the standard 10 × 10 cm^2^ reference field.

This particular LAC was found to be suitable for the measurement of dose‐area products in 6 MV beams of 5 cm diameter or less. Even when the LAC is not exactly aligned with the beams’ central axis, its response is nearly constant: With a signal drop of just 0.05% per mm of lateral misalignment, precise *DAP*_w_ measurements should therefore be achievable without a scanning water tank. Compared to point dosimeters, this would make the LAC a more practical dosimeter for routine measurements in small fields. However, users should verify this for their measurement geometry where it is different from ours, because the LAC also responds to the scattered and leakage radiation outside the primary beam. In addition, care must be taken to orient the chamber at right angles to the source in order to avoid over‐response due to the additional amount of exposed air within the sensitive volume.

The uncertainty of *N*_D,w,LAC_ was 1.3% (k = 1) when the calibration was performed in a field collimated by a 5 cm diameter cone. Although the uncertainty of *N*_D,w,LAC_ appears much reduced when the calibration is done in the standard 10 × 10 cm^2^ field, we do not recommend using those large fields for the calibration of this detector due to an observed over‐response of the LAC.

The *DAP*_w_‐based calibration method requires a relative dose measurement with high precision over two or more orders of magnitude. For a circular field, *DAP*_w_ results from profile scans with small point‐dose detectors (EFD, CC13) are consistent with those obtained with radiochromic film measurements (EBT3). Film also is useful for the measurement of *DAP*_w_ in fields of noncircular symmetry, but in any case, the results from multiple films exposed to at least two dose levels should be combined. In order to reduce the overall uncertainty further, the EBT3 response should be corrected for the changes in photon spectrum that occur in the periphery of the calibration field.

## CONFLICT OF INTEREST

No conflict of interest.
